# The types and durations of antiplatelet drugs for symptomatic vertebrobasilar artery stenosis: a retrospective study

**DOI:** 10.3389/fneur.2025.1553459

**Published:** 2025-04-03

**Authors:** Xi Liu, Guanghong Zhong, Hongli Gu, Yangchun Wen, Xiaojing Zhong, Jia Zhang, Ying Xie

**Affiliations:** ^1^Department of Neurology, Heyuan Hospital of Traditional Chinese Medicine, Heyuan, China; ^2^Department of Neurology, Heyuan People’s Hospital, Heyuan, China

**Keywords:** symptomatic vertebrobasilar artery stenosis, antiplatelet drugs, CYP2C19 gene, ticagrelor, clopidogrel

## Abstract

**Background:**

Patients with symptomatic vertebrobasilar artery stenosis not only experience a first stroke event, but also have a high risk of recurrent stroke. Even though interventional techniques have been widely used in the treatment of cerebral infarction, the therapeutic efficacy for symptomatic vertebrobasilar artery stenosis patients are not superior to that achieved with simple drug therapy. However, the optimal choice of drugs and the duration of their use in a pure drug regimen remain unclear.

**Methods:**

This retrospective study analyzed data from Heyuan People’s Hospital (2021–2023) on patients with vertebrobasilar artery stenosis. Patients were grouped by treatment duration (30/90 days ticagrelor vs. 90 days clopidogrel), all receiving aspirin. Outcomes included ischemic events, bleeding, and complications. SPSS version 22.0 was employed for statistical analysis.

**Results:**

This study included 217 patients with symptomatic vertebrobasilar artery stenosis. Clinical features and outcomes of efficacy and safety analyses were conducted. No significant differences in baseline data or safety outcomes were found. However, a significant difference in endpoint events was observed within 90 days for specific subgroups of symptomatic intracranial vertebrobasilar artery stenosis and CYP2C19 gene deletion.

**Conclusion:**

For patients with symptomatic vertebrobasilar artery stenosis, the ticagrelor plus aspirin regimen may provide an alternative therapeutic option to the aspirin plus clopidogrel bisulfate regimen. Furthermore, this regimen may represent a favored treatment choice for specific patient subpopulations.

## 1 Introduction

Posterior circulation ischemic stroke (PCIS) constitutes between 25 and 40% of all cerebral infarctions, whereas symptomatic stenosis of the vertebrobasilar artery (sVBA) represents a prominent etiology (1, 2). sVBA is characterized by transient ischemic attack (TIA) and stroke events originating from the vertebrobasilar artery system, accompanied by a stenosis severity of ≥50%. According to research conducted by British scholars, in individuals with symptomatic moderate-to-severe sVBA, the 90-day recurrence rate of stroke was 24.6%, a rate significantly elevated compared to a control group without stenosis (7.2%) (3).

To date, five randomized controlled trials (RCTs) have been conducted to investigate the CAVATAS, SAMMPRIS, VISST, VAST, and VIST trials (4–8). None of these RCTs have demonstrated that interventional vascular therapy for sVBA offers superior efficacy compared to medical therapy alone. Medical therapy remains the primary treatment option, with the standard regimen consisting of aspirin and clopidogrel (Plavix) administered for 3 months (9). However, owing to the scarcity of high-quality clinical studies on medical therapy for sVBA, there remains no consensus regarding the optimal choice and duration of medical therapy for this condition.

The present study conducted a comparative analysis of aspirin combined with ticagrelor and aspirin combined with Plavix, respectively, along with a comparison of ticagrelor usage durations of 1 versus 3 months, aiming to assess the effectiveness and safety of these treatment regimens in patients with sVBA. Furthermore, subgroup analyses were performed based on CYP2C19 genotype and the location of vascular stenosis, thereby providing comprehensive evidence to support antiplatelet therapy in sVBA, highlighting its significant practical and clinical implications.

## 2 Materials and methods

### 2.1 Patients

The retrospective study encompassed data collected from Heyuan People’s Hospital and spanning the years 2021 to 2023.

Inclusion criteria: 1. Patients with clinical diagnosis of transient ischemic attack (TIA) or cerebral infarction in the vertebrobasilar artery system, confirmed by CTA or MRA, with moderate to severe stenosis or stenosis degree of ≥50% in the vertebrobasilar artery. 2. The patient or its family agree to undergo CYP2C19 gene testing; 3. The patient signs the informed consent form; 4. The patient did not undergo endovascular treatment or surgical treatment for the vertebrobasilar artery; 5. The NIHSS score is less than 5 points.

Exclusion criteria: 1. Bleeding or other pathological brain diseases, such as tumors, cerebrovascular malformations, brain abscesses, or other common ischemic brain diseases; 2. The mRS score before randomization is greater than 2 points; 3. There is a clear indication of anticoagulation, suspected of having cardiogenic embolism, and known heart valve disease; 4. There are contraindications to the use of aspirin, clopidogrel, and ticagrelor; 5. Intracranial hemorrhage; 6. It is expected that long-term use of non-study antiplatelet agents or non-steroidal anti-inflammatory drugs that affect platelet function will be required; 7. Randomize the use of heparin or oral anticoagulant drugs for 10 days; 8. It is likely to undergo angioplasty or vascular surgery within 3 months of randomization; 9. The planned surgical or interventional procedure requires the termination of study medication; 10. Minor stroke caused by angioplasty or vascular surgery; 11. Patients with severe heart and lung diseases, with an estimated survival period of less than 3 months; 12. Women of childbearing age who have not taken contraceptive measures and have positive pregnancy test records; 13. Patients undergoing other experiments or instrumental experiments;

### 2.2 Treatment procedures

The group 1 regimen was ticagrelor 90 mg twice daily for 30 days; The group 2 regimen was ticagrelor, twice daily for 90 days; The group 3 received clopidogrel 75 mg once daily for 90 days. All experimental groups and the control group were given aspirin 100 mg once a day for 90 days.

### 2.3 Characteristic data collection

All patients were collected with demographic characteristics such as age and height, smoking, diabetes, hypertension, NIHSS score, and etiological classification after admission.

All patients in this study had blood drawn for CYP2C19 gene testing, and the testing companies included Da’an Genetic Testing Company and Jinyu Genetic Testing Company. Patients with at least two *2 or *3 alleles including 2/2, 2/3, and 3/3 are classified as “poor metabolizers”; Patients with one *2 or *3 allele, including 1/2 and 1/3, are classified as “intermediate metabolizers.” Only patients with at least one loss-of-function allele, including *2 and *3, are classified as loss-of-function carriers.

### 2.4 Outcome assessments

Efficacy analysis: the incidence of posterior circulation ischemic stroke/transient ischemic attack, myocardial infarction, or death within 30 days, 90 days, and 180 days. Safety analysis: incidence of neurological bleeding, severe peripheral system complications, and other adverse events.

### 2.5 Statistical analysis

SPSS version 22.0 was employed for statistical analysis, with measurement data presented as the mean ± standard deviation. Prior to conducting the analysis, each dataset underwent stringent tests for normality and homogeneity of variance. Based on the adherence of the data to a normal distribution, the appropriate statistical method was selected. Pre- and post-treatment differences within each group were assessed using the paired rank-sum test. Differences between the two groups were compared using the rank-sum test for two independent samples, while the chi-square test was utilized to compare treatment response rates. Statistical significance was determined at a *p*-value threshold of <0.05.

## 3 Results

### 3.1 Clinical features

**Figure 1:**
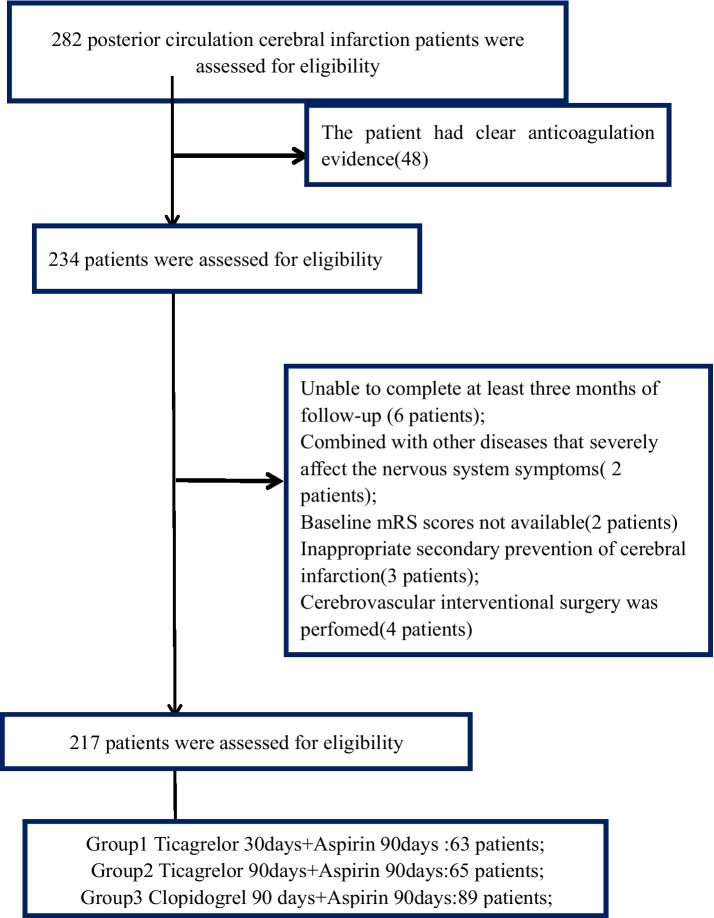
The enrollment diagram for Posterior circulation cerebral infarction patients.

A total of 217 patients with symptomatic vertebrobasilar artery stenosis who met the inclusion and exclusion criteria were included in the screening process of this study (see Figure 1). The group 1 included 63 patients, including 46 males and 17 females with an average age of 66.1 years. Among them, there were 41 cases of extracranial vertebrobasilar artery stenosis, 22 cases of intracranial vertebrobasilar artery stenosis, 40 cases of CYP2C19 gene carriers with deletions, and 23 cases of CYP2C19 gene carriers. The average NIHSS score of the admitted patients was 3.2 points, and the NIHSS score at discharge was 2.1 points. The group 2 included 65 patients, including 44 males and 21 females, with an average age of 62.3 years. Among them, there were 43 cases of extracranial vertebrobasilar artery stenosis, 22 cases of intracranial vertebrobasilar artery stenosis, 42 cases of CYP2C19 gene carriers with deletions, and 23 cases of CYP2C19 gene carriers. The average NIHSS score of the admitted patients was 3.3 points, and the NIHSS score at discharge was 1.9 points. The group 3 included 89 patients, including 67 males and 23 females with an average age of 67.5 years. Among them, there were 59 cases of extracranial vertebrobasilar artery stenosis, 30 cases of intracranial vertebrobasilar artery stenosis, 54 cases of CYP2C19 gene carriers with deletion, and 35 cases of CYP2C19 gene carriers. The average NIHSS score of the admitted patients was 3.3 points, and the NIHSS score at discharge was 2.4 points. There were no significant differences in baseline data such as hypertension history, diabetes history, coronary heart disease history, smoking and drinking history between the three groups of patients (Table 1).

**Table 1:** Differences in baseline clinical characteristics between the three groups.

	Group 1 (aspirin 90 days + ticagrelor 30 days)	Group 2 (aspirin 90 days + ticagrelor 90 days)	Group 3 aspirin (90 days + clopidogrel 90 days)	*p* value
Patients, No.	63	65	89	
Gender				0.816
Male	46	44	67	
Female	17	21	23	
Age, mean (years)	66.1 ± 14.3	62.3 ± 14.1	67.5 ± 15.2	0.546
Hypertension	27	23	41	0.558
Diabetes	29	31	38	0.806
Hyperlipidemia	48	53	71	0.748
Coronary heart disease	5	6	9	0.901
Previous stroke	42	39	53	0.670
Drinking	23	31	38	0.440
Smoking	42	39	53	0.670
Narrowing of blood vessels				0.987
Vertebral artery (extracranial segment)	41	43	59	
Vertebral arteries (intracranial segments) and basilar arteries	22	22	30	
CYP2C19 gene				0.872
CYP2C19 gene carrying	23	23	35	
CYP2C19 gene deletion	40	42	54	
Admission NIHSS score	3.2	3.3	3.3	0.673
NIHSS score at discharge	2.1	1.9	2.4	0.446

### 3.2 Outcome of efficacy analysis

In the group 1, there were 6 cases of stroke or transient ischemic attack within 30 days, including 3 cases of CYP2C19 gene deletion, 3 cases of CYP2C19 gene carriers, 2 cases of vertebrobasilar artery extracranial stenosis, and 4 cases of vertebrobasilar artery intracranial stenosis. The incidence of stroke or transient ischemic attack within 90 days was 13 cases, including 8 cases of CYP2C19 gene deletion, 5 cases of CYP2C19 gene carriers, 6 cases of vertebrobasilar artery extracranial stenosis, and 7 cases of vertebrobasilar artery intracranial stenosis.

In the group 2, there were 5 cases of stroke or transient ischemic attack within 230 days, including 3 cases of CYP2C19 gene deletion, 2 cases of CYP2C19 gene carriers, 2 cases of vertebrobasilar artery extracranial stenosis, and 3 cases of vertebrobasilar artery intracranial stenosis. The incidence of stroke and transient ischemic attack within 90 days was 11 cases, including 7 cases of CYP2C19 gene deletion, 4 cases of CYP2C19 gene carriers, 4 cases of vertebrobasilar artery extracranial stenosis, and 7 cases of vertebrobasilar artery intracranial stenosis.

In the group 3, 12 cases of stroke or transient ischemic attack occurred within 30 days, including 8 cases of CYP2C19 gene deletion, 4 cases of CYP2C19 gene carriers, 4 cases of vertebrobasilar artery extracranial stenosis, and 8 cases of vertebrobasilar artery intracranial stenosis. There were 20 cases of stroke or transient ischemic attack within 90 days, including 15 cases of CYP2C19 gene deletion, 5 cases of CYP2C19 gene carriers, 8 cases of vertebrobasilar artery extracranial stenosis, and 12 cases of vertebrobasilar artery intracranial stenosis.

**Figure 2:**
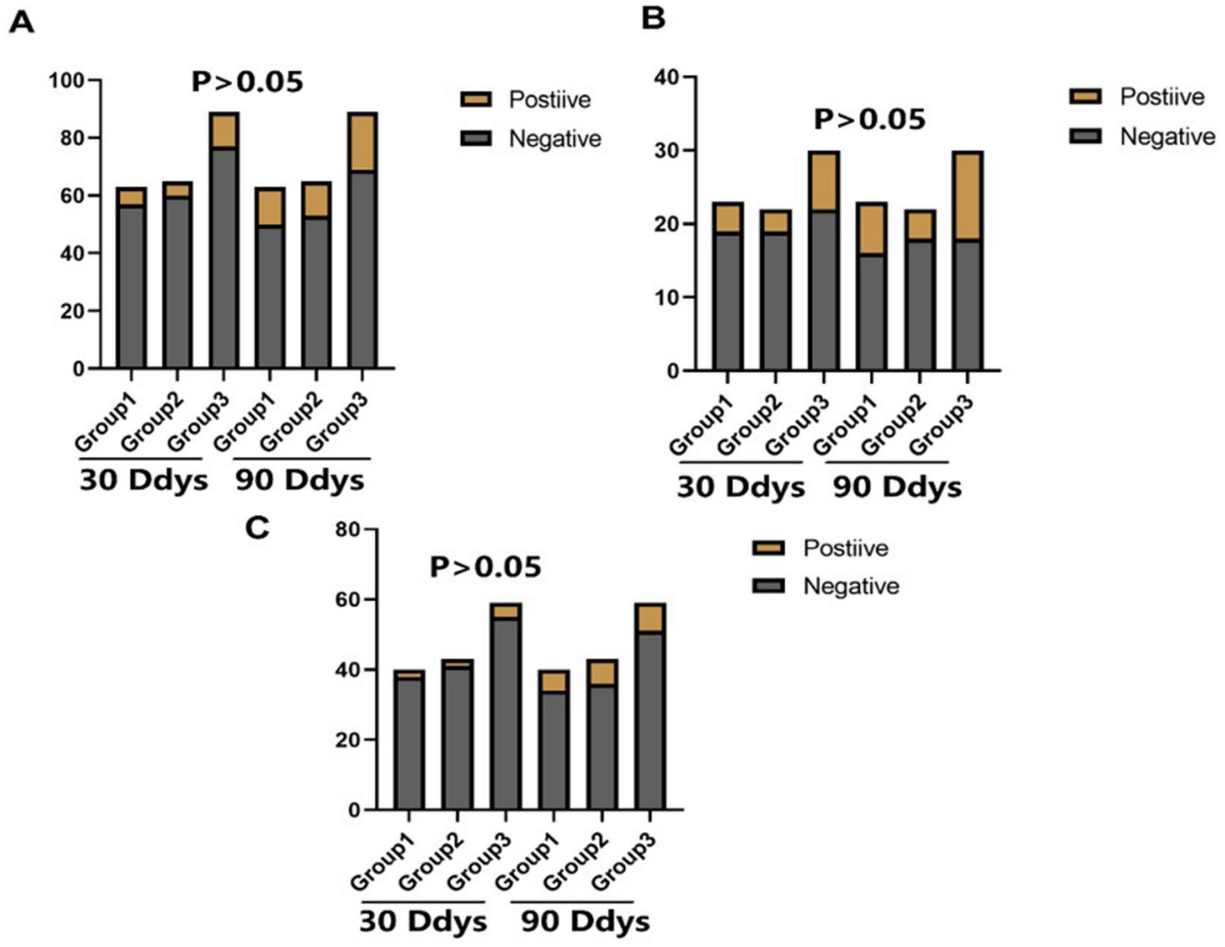
The statistical results of the incidence of end point events among the three groups. Group 1: Aspirin 90 days + ticagrelor 30 days; Group 2: Aspirin 90 days + ticagrelor 90 days; Group 3: Aspirin 90 days + clopidogrel 90 days: Positive: Stroke or TIA occurred: Negative: No stroke or TIA occurred; 30 Days: Incidence of stroke or TlA within 30 days; 90 Days: Incidence of stroke or TlA within 90 days. **(A)** The comparative of the incidence of stroke or TIA in different groups. **(B)** The comparative of the incidence of stroke or TIA in different groups in intracranicalsegments of vertebrobasilar artery. **(C)** The comparative of the incidence of stroke or TIA in different groups in extracranicalsegments of vertebrobasilar artery.

**Figure 3:**
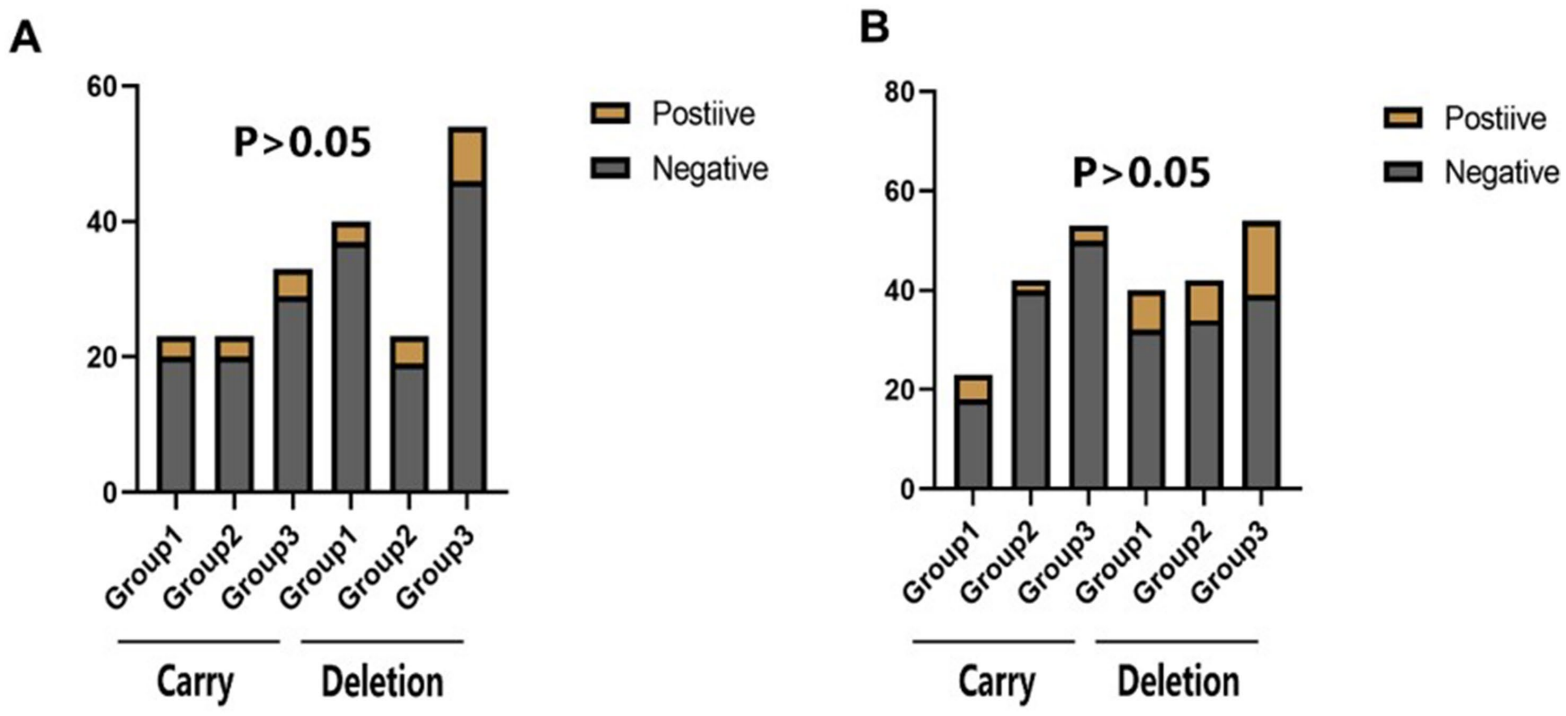
The statistical results of the incidence of end point events in the subgroup analysis of Cyp2c19 gene. Carry: Cyp2c19 gene carrier; Deletion: Cyp2c19 gene deletion. **(A**) The comparison of the incidence of stroke or TIA within 30 days of CYP2C19 genotype in different groups. **(B)** The comparison of the incidence of stroke or TlA within 90 days of CYP2C19 genotype in different groups.

**Figure 4:**
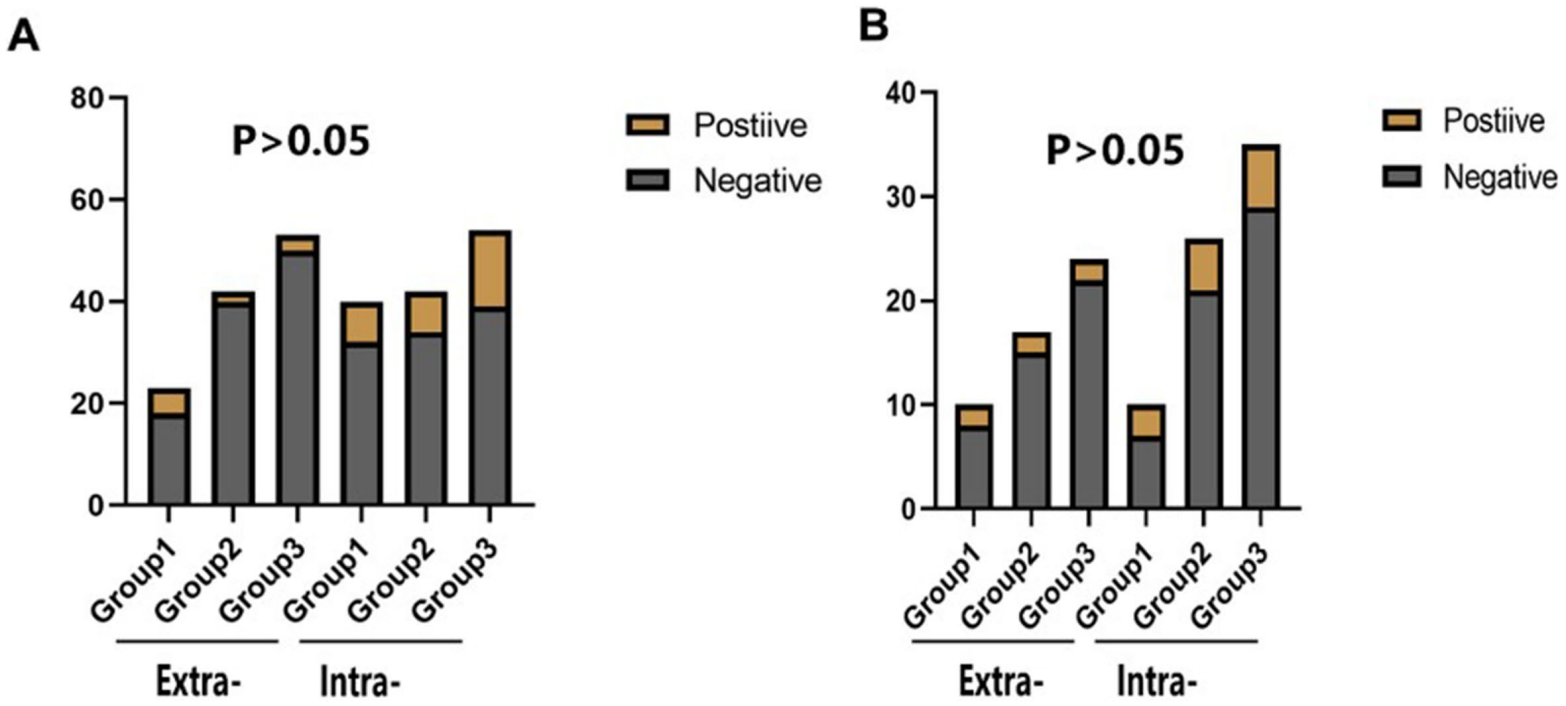
The statistical results of the incidence of end-point events in the subgroup analysis of the stenosis location of the responsible artery. Extra-: Extracranial segment of vertebrobasilar; Intra-: Intracranial segment of vertebrobasilar. **(A)** The comparison of the incidence of stroke or TIA within 30 days of different vertebrobasilarstenosis segments in different groups of CYP2C19 gene carriers. **(B)** The comparison of the incidence of stroke or TIA within 90 days of different vertebrobasilarstenosis segments in different groups of CYP2C19 gene carriers.

**Figure 5:**
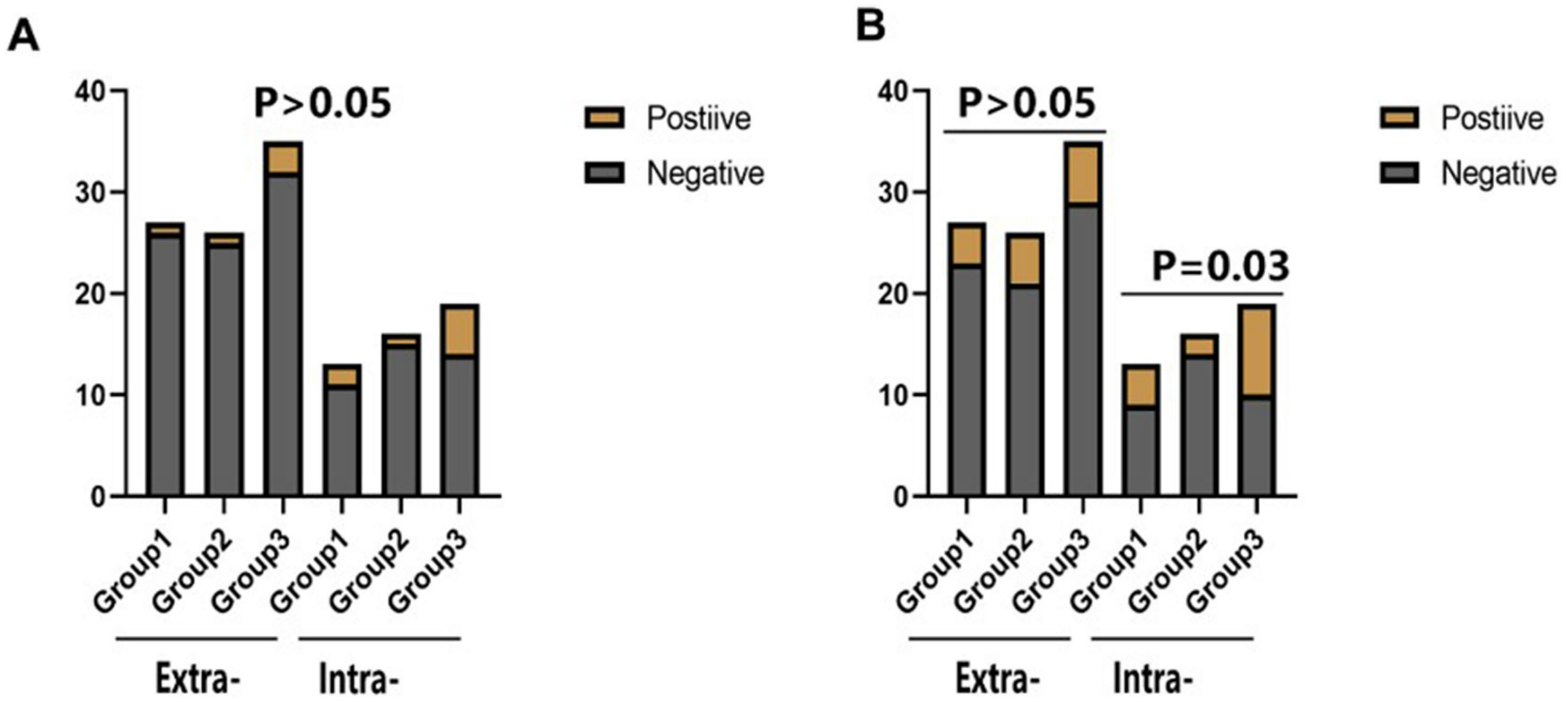
The statistical results of the incidence of end-point events in the combined subgroup analysis of the stenosis location of the responsible artery and the deletion of the Cyp2c19 gene. Extra-: Extracranial segment of vertebrobasilar; Intra-: Intracranial segment of vertebrobasilar. **(A)** The comparison of the incidence of stroke or TIA within 30 days of different vertebrobasilarstenosis segments in different groups of CYP2C19 gene deletion. **(B)** The comparison of the incidence of stroke or TiA within 90 days of different vertebrobasilarstenosis segments in different groups of CYP2C19 gene deletion.

There was no statistical difference in the incidence of endpoint events among the three groups in different subgroups at 30 days (Figures 2–4). However, in subgroup analysis, we found that there was a statistically significant difference in the three groups of drugs for patients with symptomatic intracranial vertebrobasilar artery stenosis and CYP2C19 gene deletion within 90 days (Figure 5).

### 3.3 Outcome of safety analysis

There were no significant differences in the incidence of severe bleeding, fatal gastrointestinal bleeding, cerebral hemorrhage, adverse events, mortality, and myocardial infarction between the three groups at both 30 and 90 days (Table 2).

**Table 2:** Outcome of the three groups.

	Group 1 (aspirin 90 days + ticagrelor 30 days)				Group 2 (aspirin 90 days + ticagrelor 90 days)				Group 3 aspirin (90 days + clopidogrel 90 days)			
	63				65				89			
	CYP2C19 gene carrying		CYP2C19 gene deletion		CYP2C19 gene carrying		CYP2C19 gene deletion		CYP2C19 gene carrying		CYP2C19 gene deletion	
	23		40		23		42		35		54	
Extracranial/Intracranial Segment Of Vertebrobasilar artery	13	10	27	13	17	6	26	16	24	11	35	19
Incidence of stroke (including TIA) within 30 days	3		3		3		2		4		8	
Extracranial/intracranial segment of vertebrobasilar artery	1	2	1	2	1	2	1	1	1	3	3	5
Incidence of fatal bleeding within 30 days	0		0		0		0		0		0	
Adverse events within 30 days: subcutaneous hemorrhage/dyspnea/skin rash/arrhythmia	1		0		2		1		1		1	
Incidence of stroke (including TIA) within 90 days	5		8		4		7		5		15	
Extracranial segment/intracranial segment of vertebrobasilar artery	2	3	4	4	2	2	5	2	2	3	6	9
Adverse events within 90 days, including subcutaneous hemorrhage, dyspnea, rash, and arrhythmia	2		2		4		6		2		1	
Incidence of fatal bleeding within 90 days	0		0		1		1		0		0	
Death rate	0		1		5		6		1		1	
Incidence of myocardial infarction	0		0		0		0		0		0	

## 4 Discussion

Our study demonstrates that, for patients with symptomatic vertebrobasilar artery stenosis, the aspirin plus clopidogrel regimen does not outperform the ticagrelor plus aspirin regimen. Specifically, for patients with CYP2C19 gene deficiency and symptomatic intracranial stenosis of the vertebrobasilar artery system, the combination of ticagrelor and aspirin exhibits certain advantages. Our findings align with mainstream research, indicating that ticagrelor combined with aspirin may serve as an alternative for patients who are intolerant or exhibit poor efficacy with aspirin combined with clopidogrel bisulfate (10, 11). Furthermore, our research reveals that, although a short-term combination of ticagrelor and aspirin does not yield overall benefits in terms of efficacy and safety, reducing the duration of ticagrelor use and the number of oral medications after 30 days may enhance patient experience and compliance.

Ticagrelor combined with aspirin can replace the aspirin plus clopidogrel bisulfate regimen and may even offer therapeutic advantages in specific patient populations. The underlying reasons for this are not entirely clear but may be attributed to the following:

Firstly, ticagrelor shares a similar mechanism of action with clopidogrel bisulfate, inhibiting the ADP (adenosine diphosphate) receptor in platelets, thereby reducing platelet aggregation and adhesion (12). Given the comparable inhibitory effect on platelet aggregation between ticagrelor and clopidogrel hydrogen sulfate, ticagrelor is currently also utilized as an antiplatelet agent for the prevention of cerebral infarction (13).

Secondly, the pharmacological mechanisms of the two drugs differ. Clopidogrel is a prodrug that requires hepatic cytochrome P450-mediated conversion to its active metabolite, a process that may be influenced by CYP2C19 gene polymorphism (11, 13, 14). In contrast, ticagrelor is a reversible oral antagonist that directly blocks the platelet P2Y12 receptor, and its antiplatelet effect does not necessitate metabolic activation (12, 15). Current consensus suggests that clopidogrel exhibits a limited secondary prevention effect on stroke in carriers of CYP2C19 loss-of-function alleles (16).

Our research results indicate that there are differences in the treatment effects of intracranial and extracranial stenosis in patients with CYP2C19 loss of function in the symptomatic vertebrobasilar artery system when comparing the clopidogrel bisulfate regimen with the ticagrelor regimen. This finding may be attributed to the bias inherent in our sample size. Due to the scarcity of relevant research and literature, we are unable to explore the underlying causes and mechanisms.

The limitations of this study include: (1) It is a single-center study with a relatively small sample size; (2) There were no restrictions on the use of other medications, which may have introduced bias into the results; (3) The follow-up duration was brief, and no data comparisons were conducted for patients discharged beyond 90 days; (4) It is a retrospective study, providing low-level evidence from an evidence-based medicine perspective and may be biased.

## 5 Conclusion

For patients exhibiting symptomatic vertebrobasilar artery system stenosis, the combination therapy of ticagrelor and aspirin can serve as a viable alternative to the regimen of aspirin plus clopidogrel bisulfate, particularly for specific patient subsets, including those with a deficiency in the CYP2C19 gene, individuals with intracranial stenosis within the symptomatic vertebrobasilar artery system, patients who have extensive medication experience, or those with poor adherence to treatment protocols. However, The specific type and duration of antiplatelet therapy should be individualized, determined through a collaborative decision-making process between the patient and clinician, with comprehensive consideration of the patient’s risk of recurrent ischemic events, bleeding risk, and other relevant factors. Finally, further validation through multi-center, randomized controlled trials with expanded sample sizes and extended follow-up durations is necessary to substantiate this conclusion and offer robust guidance for the clinical management of symptomatic vertebrobasilar artery stenosis.

## Data Availability

The original contributions presented in the study are included in the article/supplementary material, further inquiries can be directed to the corresponding author.
